# Nonverbal cues to deception: insights from a mock crime scenario in a Chinese sample

**DOI:** 10.3389/fpsyg.2024.1331653

**Published:** 2024-02-09

**Authors:** He Li, Hu Song, Menghan Li, Hanxue Li

**Affiliations:** ^1^School of Public Administration, Northwest University, Xi’an, China; ^2^College of Education, Hunan First Normal University, Changsha, China

**Keywords:** nonverbal cues, Asian culture, mock crime, self-adaptors, gaze aversion

## Abstract

Nonverbal behaviors could play a crucial role in detecting deception, yet existing studies on deception cues have largely centered on Western populations, predominantly university students, thus neglecting the influence of cultural and sample diversity. To address this gap, our study explored deception cues within an Asian cultural setting, utilizing a mock crime paradigm. Our sample comprised Chinese participants, including both men and women with various socioeconomic status (SES) backgrounds. Our findings revealed that compared to truth tellers, liars exhibited heightened emotions and an increased cognitive load. Furthermore, liars showed a higher frequency of self-adaptors and a longer duration of gaze aversion. Our findings contribute to a more profound understanding of deception cues within Asian culture and have implications for practical fields such as criminal interrogation.

## Introduction

Deception, which is defined as the intentional act of misleading others (DePaulo et al., 2003), is a widespread phenomenon. For instance, people involved in deceitful behavior in 14% of emails, 27% of face-to-face interactions, and 37% of telephone conversations (Hancock, 2007; Porter and ten Brinke, 2010). Despite its prevalence, previous studies have shown that people are not good at detecting deception, with an average accuracy of slightly above chance level (Bond and DePaulo, 2006). To improve deception detection accuracy, obvious and easily observable cues to deception are necessary (Hartwig and Bond, 2011; Chan et al., 2016).

### Nonverbal cues to deception

Cue theories form the theoretical basis for research on deception cues, asserting that differences in mental processes, including emotion, cognitive load, and attempted control, between liars and truth tellers can manifest as observable cues in deception detection (Levine, 2015). From the perspective of emotion, Ekman and Friesen (1969) proposed the nonverbal leakage hypothesis. This hypothesis suggests that individuals may display nonverbal behaviors, indicative of emotions such as anxiety, fear, or “duping delight,” when attempting to deceive others. Furthermore, Vrij et al. (2019) emphasized cognitive differences between liars and truth-tellers, suggesting that individuals experience a greater cognitive load during lying, manifesting as observable cues to deception. Additionally, the attempted control approach posits that individuals attempt to control their behaviors in order to appear as honest as possible during lying (Granhag and Strömwall, 2002).

Empirical studies based on cue theories have explored nonverbal cues to deception, though findings have been inconsistent (DePaulo et al., 2003; Luke, 2019). For instance, Ekman et al. (1991) investigated emotional cues of deception and observed significant differences in facial expressions between liars and truth tellers, with liars displaying fewer Duchenne smiles and more mask smiles. Mann et al. (2002) analyzed videos of criminal suspects being interrogated and found that suspects blinked less frequently while lying. Markey et al. (2022) analyzed deception cues during 911 homicide calls, finding that liars exhibited more emotional cues, such as nervousness, worry, and feeling overwhelmed. Furthermore, from a cognitive perspective, Vrij et al. (1996) instructed subjects to undergo interrogation in both truthful and deceptive scenarios and observed a significant decrease in leg and foot movements as well as hand and finger movements during deception, indicative of a greater cognitive load. Frosina et al. (2018) investigated the effects of cognitive load on nonverbal behavior during interrogation, finding that lying increased blink rate while decreasing hand gestures. Additionally, Greene et al. (1985) discovered that inhibitory control processes lead to a reduction in hand movements, leg and foot movements, and illustrators when individuals are lying.

A meta-analysis of 158 deception cues from earlier studies revealed only a few cues associated with deception, such as appearing nervous, tense, and uncooperative. Notably, these cues generally exhibited small effect sizes (median *d* = 0.10) (DePaulo et al., 2003; Wright Whelan et al., 2014). Overall, no “Pinocchio’s nose” — a clear indicator of lying — has been identified in deception detection.

### Moderators of nonverbal cues to deception

Past research has suggested that cues to deception are faint and unreliable (DePaulo et al., 2003). This may be attributed to the fact that nonverbal cues associated with deception are influenced by various variables, including situational factors and interpersonal differences (Vrij, 2008).

The influence of situational factors, such as the motivation of the liar, the complexity of the lie, and the stakes involved, on deception cues has been extensively investigated. DePaulo et al. (1983) observed that highly motivated liars are likely to manage their verbal cues more effectively than nonverbal cues, leading to an increase in the leakage of nonverbal cues compared to less motivated liars. The complexity of a lie also plays a crucial role. Vrij and Heaven (1999) found that as lying becomes more mentally demanding, liars show more indicators of cognitive load, such as speech hesitations, than truth tellers. Additionally, the stakes associated with a lie can influence nonverbal cues to deception. In high-stakes situations, liars may experience stronger emotions or increased cognitive load compared to low-stakes situations, resulting in more displays of disgust and fewer displays of sadness, as well as increased gaze aversion and head shaking during high-stakes deception (Ten Brinke and Porter, 2012; Wright Whelan et al., 2014). Meta-analysis studies have supported these findings, affirming the influence of motivational factors, stakes, and the content of deception, etc. on behavioral differences between liars and truth tellers (DePaulo et al., 2003; Sporer and Schwandt, 2006, 2007).

Interpersonal differences related to an individual’s personality, ethnicity or culture, and gender also significantly influence deception cues (Vrij, 2008). Personality traits from the Dark Triad, particularly Machiavellianism and psychopathy, are substantially associated with deception. For instance, Exline et al. (1970) found that highly Machiavellian individuals, who typically are less prone to guilt and more likely to engage in strategic self-presentation, maintain more eye contact when lying than those with lower Machiavellian traits. Psychopathic offenders, known for their superior interpersonal skills compared to their non-psychopathic counterparts, display increased head movements, provide more appropriate details, and make spontaneous corrections during deception (Klaver et al., 2007; Lee et al., 2008). Research has also highlighted the impact of cultural factors; Bond et al. (1990) discovered that Jordanians exhibited more speech pauses when lying than when telling the truth, whereas Americans did not exhibit this difference. Further, Burgoon et al. (2021) found that the behaviors of interviewees engaged in deceptive communication, such as eye blinks, hand shrugs, and vocal pitch, varied depending on their cultural orientations. Gender differences, though limited in findings, are still noteworthy. For instance, Cody and O’Hair (1983) found that in prepared lies, men suppressed the use of illustrators and exhibited more facial adaptors than women. O’Hair and Cody (1987) further revealed gender-based differences in vocal stress, with women displaying higher levels of vocal stress during prepared deception.

### The present study

Extensive research on deception cues has been conducted (DePaulo et al., 2003; Vrij et al., 2019); however, further investigation is still needed. First, existing studies have suggested the influence of culture on behavioral cues associated with deception, yet the majority of these studies were conducted in Western countries. For instance, DePaulo et al.’s (2003) meta-analysis reviewed 120 studies on behavioral cues to deception, of which 117 studies were conducted in Western countries, with only three in Asian countries (Japan and Jordan). This overlooks cultural variations in nonverbal cues associated with deception. Second, a large proportion of previous studies on deception cues has involved university students as participants. A recent study explored nonverbal cues to deception using Singaporean non-student participants, but the sample exclusively consisted of men (Chan et al., 2016). This lack of diversity in samples is a concern, as interpersonal differences such as gender and soicoeconomic status (SES) can significantly affect emotions and cognition (Kraus et al., 2012; Hyde, 2014; Manstead, 2018), and potentially, deception cues.

The present study aimed to enrich our understanding of deception cues within an Asian context by involving Chinese non-student participants with diverse SES, including both men and women, in a mock crime scenario. By controlling for interpersonal differences such as gender and SES, we sought to provide a more nuanced examination of deceptive behaviors. The focus of our investigation was primarily on nonverbal cues commonly linked with deception, such as blinks, gaze aversion (frequency and duration), head movements, self-adaptors, illustrators, hand and finger movements, trunk movements, and foot and leg movements.

We developed and tested predictions concerning the effects of veracity on participants’ mental states and nonverbal cues, based on cue theories. Our predictions were as follows:

(1) Emotional intensity, cognitive load, and attempted control:

All cue theories of deception predict that liars would exhibit more intense emotions, a higher cognitive load, and greater attempted control compared to truth-tellers.

(2) Self-adaptors:

Emotion theories of deception predict an increase in emotion-related cues such as self-adaptors among liars. Conversely, the cognitive approach posits that liars, burdened by a higher cognitive load, will exhibit a decrease in self-adaptors. Additionally, the attempted control perspective also suggests a reduction in self-adaptors as liars attempt to inhibit their behavior to conceal deception.

(3) Gaze aversion:

Both emotional and cognitive theories of deception predict increased gaze aversion in liars, due to intensified emotions and cognitive overload. However, the attempted control perspective suggests a reduction in gaze aversion, as liars try to counter the common belief that gaze aversion is a primary deception cue (The Global Deception Research Team, 2006).

(4) Illustrators and body movements:

The cognitive approach will predict fewer illustrators and body movements (e.g., head, trunk) due to the cognitive demanding of lying. Similarly, the attempted control perspective hypothesizes a reduction in these behaviors, as liars try to inhibit their behavior to prevent the leakage of deception cues.

## Materials and method

### Participants

Fifty participants (24 women; 34.72 ± 4.69 years) from various SES were recruited and compensated for their participation in the study. Prior to the experiment, all participants provided informed consent and completed questionnaires about their demographics. Objective measures of SES, including education, occupation, and annual income, were collected for this study. The measurement questionnaire was from Chen and Zhao’s (2017) study (see Supplementary material S1 for details).

The participants were categorized into two groups based on their SES scores: 26 participants in the lower SES group (12 women) and 24 participants in the higher SES group (12 women). The SES score for each participant was determined by summing their standardized scores for education, occupation, and income. A t-test revealed a significant difference in SES scores between the lower SES group (*M* = −2.52, SD = 0.94) and the higher SES group (*M* = 2.73, SD = 1.05), *t*(48) = 18.64, *p* < 0.001.

### Materials

The stimulus materials were videotapes generated by the mock crime paradigm previously employed by Vrij et al. (2010).

Participants from higher and lower SES were randomly assigned to either the lying or truthful condition. There were 25 participants in the lying condition, comprising 11 women and 12 from higher SES. Similarly, there were 25 participants in the truthful condition, with 13 women and 12 from higher SES. Subsequently, each participant engaged in a task involving a mock crime scenario tailored to their assigned condition, followed by an interrogation.

In the truthful condition, participants engaged in a staged event in an activity room. They played a poker game with a male confederate who posed as another participant. During the game, they were interrupted three times: first, the experimenter entered the room to close the windows; second, the male confederate went outside to answer a phone call for about a minute; third, a female confederate entered the room and claimed that her wallet, which she found in the room, was missing 200RMB (about 25€). Although both participants denied taking the money, they were informed that they would be interviewed regarding the lost money.

In the lying condition, participants did not participate in the staged event. Instead, they were instructed to enter the room, take 200RMB from the wallet, and hide the money on their person. They were then shown a document that described the staged event in the truthful condition, and were told to deny taking the money during the interrogation and to pretend that they had participated in the activity described in the document. The content of the document read as follows:

“You entered a room where you found another male participant, and together you played a game of poker across a table. During the game, the experimenter entered the room, closed the windows, and left. After a while, the other participant’s mobile phone rang, and he left the room to answer the call, leaving you alone in the room for about a minute. When the other participant returned, you resumed playing poker, and then a woman entered the room looking for her wallet. She claimed that 200RMB was missing from her wallet, which she had found in the room. Both you and the other participant denied knowing anything about the missing money. The experimenter then returned to the room and informed both of you would be interviewed”.

Participants in both conditions were motivated to convince the interviewer of their innocence, as they were told that if the interviewer was convinced, they would receive an additional 20RMB (about 2.5€) for their participation. Otherwise, they would have to write a detailed statement about what happened.

Participants were then brought to an interrogation room where they were seated in front of an interviewer who wore a police uniform. The interviewer was a graduate student majoring in law and was unaware of the participant’s condition (truthful or lying) beforehand. The interrogation began with the statement, “200RMB was lost in the activity room, and I have to find out whether or not it was you who took the money. Please answer questions honestly.” Participants were then asked questions about their basic information (i.e., name, age, and ethnicity), followed by a request to describe in detail what happened in the activity room, including what they did, said, and information about people who entered the room. All the interrogations were videotaped using an EOS 5D Mark III camera positioned in front of the participants.

After the interrogation, the participants were brought to another room to fill out a questionnaire. The questionnaire comprised of two parts. The first part of the questionnaire was related to manipulation checks of the participant’s motivation to convince the interviewer, and perceived likelihood of positive and negative consequences of the interrogation. Specifically, the items were: (1) To what extent did you attempt to convince the interviewer during the interrogation? (2) How likely did you think it was that you would receive the 20RMB? (3) How likely did you think it was that you would be required to provide a detailed statement? The second part of the questionnaire contained items pertaining to the participant’s experiencing emotions, cognitive load, and attempted control. Emotions were measured with three items: (1) To what extent did you feel nervous during the interrogation? (2) To what extent did you feel guilty during the interrogation? (3) To what extent did you feel anxious during the interrogation? Cognitive load was measured with three items too, which were: (1) To what extent did you find the interview mentally difficult? (2) To what extent did you have to think hard during the interrogation? (3) To what extent did you have to concentrate during the interrogation? Attempted control was measured with one item: To what extent did you make an effort to speak in a convincing manner during the interrogation? All items of the questionnaire were rated on 5-point Likert scales, in line with previous deception research (Chan et al., 2016).

Upon completion, the participants were informed that they had successfully convinced the interviewer, and each of them received the additional payment of 20RMB. Finally, the participants were debriefed and thanked for their participation.

### Design

In the present study, veracity (lying vs. truth-telling) served as the independent variable. Control variables comprised gender (man vs. woman) and SES (higher SES vs. lower SES). The dependent variables included participants’ self-reports of mental states and the nonverbal cues exhibited during the mock interrogation.

#### Coding of nonverbal behavior

The segments of the mock interrogation videotapes, in which participants described their experience in the activity room to convince the interviewer, were used for the analysis of nonverbal cues. The average length of these segmented videotapes was 1.05 ± 0.43 min. The following nonverbal cues were coded:

Blinks: frequency of both eyes being briefly closed at the same time.

Gaze aversion (duration): the number of seconds that the participant did not maintain eye contact with the interviewer.

Gaze aversion (frequency): the number of times that the participant broke eye contact with the interviewer.

Head movements: the frequency of visible head movements, including vertical or horizontal movements. A continuous quick head shaking or nodding was coded as a head movement.

Self-adaptors: the frequency of movements in which one hand is in contact with the other hand or other parts of the body or face, such as scratching the head, picking the nose, and grasping the wrist. It occurs with the purpose of satisfying self needs or body needs, or coping with emotions. A short succession of quick scratches was scored as one movement.

Illustrators: the frequency of hand and arm movements that accompany speech and serve to amplify, complement, or modify what is being said. For instance, individuals may employ the gap between their two palms to indicate the size or height of an object when describing it.

Hand and finger movements: the frequency of movements of hand and fingers, excluding any movements involving the arm. Fingers that happened to move simultaneously were scored as one movement.

Trunk movements: the frequency of visible forward, backward, or sideward movements of the torso. Any clearly noticeable changes in seated position were also recorded and scored.

Foot and leg movements: the frequency of movements involving the feet and legs. A brief sequence of leg shakes was counted as a single movement. In cases of prolonged leg shakes, each second of continuous shaking was scored as one movement.

Two trained undergraduates, who were blind to the experimental hypotheses, were recruited to perform the coding work. They independently coded all the segments of video recordings. For cues such as self-adaptors and illustrators, coding was done with audio input, while for other cues, coding was done without audio input. This approach was chosen because self-adaptors are linked to emotional regulation or self-satisfaction, and illustrators serve as supplementary expressions to verbal communication. The inclusion of audio input facilitated a better understanding of participants’ behaviors, ensuring coding accuracy and precision.

Upon analyzing the coded data, it was observed that trunk movements were rarely observed during the interrogation. Therefore, no further analysis was conducted on trunk movements. Inter-rater reliability was calculated for the remaining coded cues. As depicted in Table 1, all coded cues, except for hand and finger movements (*r* = 0.56, *ICC* = 0.56), exhibited good inter-rater reliability (*r*s > 0.80; *ICC*s > 0.75). Consequently, hand and finger movements were excluded from subsequent analyses. The average scores derived from the two coders were used as the scores for the nonverbal cues. Furthermore, all nonverbal cues were adjusted for the length of the interrogation, that is, they were calculated as the duration or frequency of specific movements per minute.

**Table 1:** Inter-rater reliability for all the coded nonverbal cues.

Variables	Pearson correlations	Intra-class coefficients
Blinks	0.964^**^	0.960
Gaze aversion (frequency)	0.845^**^	0.829
Gaze aversion (duration)	0.856^**^	0.794
Head movements	0.862^**^	0.819
Self-adaptors	0.813^**^	0.796
Illustrators	0.955^**^	0.907
Hand and finger movements	0.561^**^	0.559
Foot and leg movements	0.946^**^	0.922

^**^p < 0.01.

### Statistical analysis

First, correlation analyses were conducted to examine the relations between variables such as participants’ gender and SES, and their mental states, and nonverbal cues. Then, univariate analyses, employing a General Linear Model, were conducted to assess the main effects of veracity, including gender and SES as covariates. Statistical analyses were performed using SPSS 26 software. Additionally, effect size and statistical power calculations were performed using G*Power 3.1.9 (Faul et al., 2007).

## Results

### Manipulation checks

First, we conducted an investigation to determine the effectiveness of our motivation manipulation. Our results revealed that 84% of participants considered their level of motivation convincing, scoring 3 or higher on the five-point Likert Scale. This indicates that the majority of participants were motivated to persuade the interviewer successfully. Furthermore, we examined whether participants’ veracity (lying vs. truth-telling) and gender (man vs. woman) were independent for those who rated their motivation as 3 or higher. Our analysis revealed no significant gender effect on truth-tellers and liars’ motivation to be convincing, $\chi^{2}$ = 0.10, *p* = 0.76. Similarly, SES was not a significant factor affecting the motivation of truth-tellers and liars to be convincing, $\chi^{2}$ = 0.39, *p* = 0.53.

Next, we evaluated participants’ perceptions of the potential consequences of the interrogation. Regarding the likelihood of receiving a 20RMB incentive, 86% of the participants rated it as 3 or higher on the five-point Likert Scale. We then examined whether the participants’ veracity and gender were independent for those who rated the likelihood of receiving this incentive as 3 or higher. The results showed no significant gender effects on the perceived likelihood of receiving 20RMB among truth-tellers and liars, $\chi^{2}$ = 0.03, *p* = 0.86. Similarly, SES did not significantly affect their perceptions, $\chi^{2}$ = 0.03, *p* = 0.86. On the other hand, 82% of the participants rated the likelihood of receiving a punishment (writing a statement) as less than 3 on the scale. We found that neither gender nor SES affected the perceptions of receiving a punishment among truth-tellers and liars who rated the likelihood as 3 or less ( $\chi^{2}$ = 0.03, *p* = 0.87; $\chi^{2}$ = 0.02, *p* = 0.90).

Overall, these results suggest that participants are motivated to be convincing in the mock interrogation. Furthermore, the perceived consequences of the interrogations for both truth-tellers and liars were not affected by their gender or SES.

### Correlation analyses

Prior to conducting the correlation analyses, we assessed the reliability of items measuring emotion and cognitive load. The three emotion items exhibited acceptable reliability (Cronbach’s alpha = 0.75), while the three cognitive load items demonstrated questionable reliability (Cronbach’s alpha = 0.53). Thus, the three emotion items were combined to create a composite “emotion” index, while the three cognitive load items were analyzed individually.

Then, correlation analyses were conducted. The results showed no significant correlations between participants’ gender and their reported mental states. However, a significant correlation was found between participants’ gender and the duration of gaze aversion (*r* = 0.335, *p* < 0.05). This suggests that women may exhibit a longer duration of gaze aversion compared to men. This gender difference could be attributed to the interviewer in the mock interrogation being a man. It is plausible that individuals might express a longer duration of gaze aversion when interacting with someone of the opposite sex.

Regarding SES, it was negatively correlated with emotions (*r* = −0.375, *p* < 0.01), perceived mental difficulty (*r* = −0.285, *p* < 0.05), and concentration levels (*r* = −0.422, *p* < 0.01). This indicated that individuals with lower SES tend to experience more intense emotions and had a greater cognitive load than those with higher SES. However, SES did not show a significant correlation with nonverbal cues.

Further, our analyses revealed a positive correlation between concentration and the duration of gaze aversion (*r* = 0.328, *p* < 0.05), while no other significant correlations were found between mental states and nonverbal cues. These results were shown in Table 2.

**Table 2:** Correlations between gender, SES, and mental states, and nonverbal cues.

	1	2	3	4	5	6	7	8	9	10	11	12	13
1. Gender													
2. SES	0.038												
3. Emotions	0.137	−0.375^**^											
4. Mental difficulty	0.221	−0.285^*^	0.514^**^										
5. Thinking hard	0.002	−0.262	0.294^*^	0.446^**^									
6. Concentration	−0.031	−0.422^**^	0.220	0.129	0.223								
7. Attempted control	−0.044	0.109	0.294^*^	−0.082	0.006	−0.099							
8. Blinks	0.092	−0.231	0.165	−0.010	−0.125	0.053	−0.010						
9. Gaze aversion (frequency)	0.059	−0.202	−0.053	−0.028	−0.176	0.242	0.054	−0.070					
10. Gaze aversion (duration)	0.335^*^	−0.123	0.174	0.144	0.006	0.328^*^	−0.061	−0.049	0.446^**^				
11. Head movements	−0.004	−0.265	0.109	0.033	−0.163	−0.008	−0.103	0.072	0.216	0.297^*^			
12. Adaptors	0.127	−0.009	0.159	0.218	0.074	−0.049	0.056	−0.061	−0.136	0.248	0.030		
13. Illustrators	−0.052	−0.100	−0.074	−0.081	−0.058	0.022	−0.033	−0.241	−0.062	0.127	0.208	−0.018	
14. Foot and leg movements	−0.193	−0.076	0.261	0.212	0.212	0.086	−0.256	−0.203	0.191	0.166	0.208	0.276	−0.101

^*^p < 0.05; ^**^p < 0.01.

### Univariate analyses on liars’ emotion, cognitive load, and attempted control

Table 3 presents the descriptive statistics for emotions, cognitive load, and attempted control as a function of veracity.

**Table 3:** Emotion, cognitive load, and attempted control as a function of veracity.

Variables	Lying		Truth-telling	
	*M*	SD	*M*	SD
Emotion	2.71	0.83	1.85	0.69
Mental difficulty	2.48	0.87	2.00	1.00
Thinking hard	2.48	0. 96	2.60	1.19
Concentration	3.68	0.99	3.88	0.88
Attempted control	2.92	0.95	2.76	1.16

To test Hypothesis 1, we conducted univariate analyses to examine differences in emotions, cognitive load, and attempted control between liars and truth tellers. We initially included gender and SES as covariates in these analyses. Our findings revealed a significant main effect of veracity on emotions. Liars experienced more intense emotions (*M* = 2.71, SD = 0.83) compared to truth tellers (*M* = 1.85, SD = 0.69), *F*(1, 46) = 20.76, *p* < 0.001, $\eta_{p}^{2}$ = 0.31. A post-hoc power analysis with an effect size of $\eta_{p}^{2}$ = 0.31 and an alpha level of 0.001 yield a statistical power of 0.88.

Regarding cognitive load, we observed a significant effect of veracity in reported mental difficulty. Liars (*M* = 2.48, *SD* = 0.87) reported that they experienced greater mental difficulty than truth tellers (*M* = 2.00, *SD* = 1.00), *F*(1, 46) = 4.31, *p* < 0.05, $\eta_{p}^{2}$ = 0.09. The post-hoc power analysis for this effect, using the effect size $of\;\eta_{p}^{2}$ = 0.09 and an alpha level of 0.05, resulted in a statistical power of 0.59. However, no significant effect of veracity on attempted control was found.

We also conducted univariate analyses on the effects of veracity without incorporating covariates such as gender and SES. The results largely supported our initial findings, demonstrating that liars experienced more intense emotions than truth-tellers, albeit with a reduced effect size, *F*(1, 48) = 15.68, *p* < 0.001, $\eta_{p}^{2}$ = 0.25. However, when the covariates were excluded, the effects of veracity on mental difficulty was not significant any more, *F*(1, 48) = 3.27, *p* = 0.077, $\eta_{p}^{2}$ = 0.06. These discrepancies indicate the potential influences of interpersonal factors such as gender and SES on the relations between veracity and mental states.

### Univariate analyses on nonverbal cues to deception

Table 4 shows the descriptive statistics for the coded nonverbal cues as a function of veracity.

**Table 4:** Nonverbal cues as a function of veracity.

Variables	Lying		Truth-telling	
	*M*	SD	*M*	SD
Blinks	43.82	18.64	38.32	21.02
Gaze aversion (frequency)	8.52	4.43	8.92	4.80
Gaze aversion (duration)	0.28	0.20	0.21	0.13
Head movements	8.83	4.86	7.01	4.66
Self-adaptors	6.48	7.62	3.17	3.21
Illustrators	8.16	12.08	8.69	11.68
Foot and leg movements	6.02	9.81	5.48	9.05

In testing Hypotheses 2, 3, and 4, we employed univariate analyses to examine the effects of veracity on nonverbal cues, including blinks, gaze aversion (both frequency and duration), head movements, self-adaptors, illustrators, and foot and leg movements. In these analyses, gender and SES were employed as covariates. Our findings showed a significant main effect of veracity on self-adaptors. Specifically, liars were found to use more self-adaptors (*M* = 6.48, SD = 7.62) compared to truth tellers (*M* = 3.17, SD = 3.21), *F*(1, 46) = 4.26, *p* < 0.05, $\eta_{p}^{2}$ = 0.09. A post-hoc power analysis, utilizing the effect size of $\eta_{p}^{2}$ = 0.09 and an alpha level of 0.05, showed an achieved power of 0.59. Additionally, there was a marginally significant main effect of veracity on the duration of gaze aversion, with liars (*M* = 0.28, SD = 0.20) showing a longer duration than truth-tellers (*M* = 0.21, SD = 0.13), *F*(1, 46) = 3.83, *p* = 0.057, $\;\eta_{p}^{2}$ = 0.08. The post-hoc power analysis, with an effect size of $\;\eta_{p}^{2}$ = 0.08 and an alpha level of 0.05, showed an achieved power of 0.53. No other significant effects of veracity were found.

Further analyses were conducted without the inclusion of covariates such as gender and SES. These analyses revealed a marginally significant effect of veracity on self-adaptors, *F*(1, 48) = 4.00, *p* = 0.051, $\eta_{p}^{2}$ = 0.08. However, the exclusion of covariates resulted in the effect of veracity on the duration of gaze aversion becoming statistically non-significant, *F*(1, 48) = 2.69, *p* = 0.107, $\eta_{p}^{2}$ = 0.05. These variations suggest the potential influence of gender and SES on the relations between veracity and nonverbal cues.

## Discussion

The present study investigated mental states and nonverbal cues associated with deception in a mock crime scenario, utilizing a Chinese sample comprising both men and women with various SES. Our focus on controlling interpersonal differences such as gender and SES allowed for a more nuanced examination of nonverbal cues to deception. The findings revealed that liars experienced more intense emotions, specifically nervousness, guilt, and anxiety, and encountered a greater cognitive load, termed mental difficulty, than truth-tellers. Furthermore, liars exhibited a higher frequency of self-adaptors and a longer duration of gaze aversion compared to truth-tellers.

The finding that liars exhibit more intense emotions and greater mental difficulty than truth-tellers supports both the emotional and cognitive perspectives. From an emotional perspective, it is believed that liars experience increased fear and anxiety due to the concern of their deception being discovered, coupled with feelings of guilt from their dishonest actions (DePaulo et al., 2003). Additionally, the cognitive theory suggests that lying involves suppressing truthful information, fabricating falsehoods, and regulating one’s behavior, all leading to an increased cognitive load (Walczyk et al., 2014). These emotional and cognitive disparities between liars and truth-tellers form the basis for exploring behavioral indicators of deception. However, our study did not find differences in attempted control between liars and truth-tellers. This may be because attempted control, often associated with experienced liars (Ten Brinke and Porter, 2012), is not a strategy frequently employed by ordinary people.

The increased frequency of self-adaptors among liars aligns with the emotional prediction. While our study observed that liars experience a higher cognitive load, termed mental difficulty, than truth-tellers, the finding of increased self-adaptors contrasts with the prediction derived from the cognitive approach. This suggests that the cognitive load in our study might not have been strong enough to elicit behavior changes, implying that emotional factors could play a more dominant role in the expression of self-adaptors during deception. Furthermore, our result corresponds with prior research on emotionally deceptive scenarios (Porter et al., 2008), demonstrating that self-adaptors may serve as an emotional indicator of deception. However, it is essential to acknowledge studies using the mock crime paradigm that reported no discernible difference in self-adaptors between liars and truth-tellers (Vrij et al., 2008; Burgoon et al., 2015; Chan et al., 2016). This discrepancy may be attributed to factors such as the content of deception, stakes involved, preparation, participant samples, etc. (Gerlach et al., 2019; Şen and Küntay, 2019; Vrij et al., 2019).

Regarding gaze aversion, our finding of a longer duration among liars supports both emotional and cognitive perspectives. Previous studies have suggested that both intensified emotional experience and heightened cognitive load contribute to more expressions of gaze aversion (DePaulo et al., 2003; Doherty-Sneddon and Phelps, 2005; Mann et al., 2012). In our mock interrogation, liars experienced more intense emotions and greater mental difficulty, which likely led to their prolonged duration of gaze aversion. Nevertheless, it is worth noting that previous studies have suggested no difference in gaze aversion between liars and truth-tellers (Mann et al., 2012, 2013). This could occur because liars might deliberately increase eye contact, either to convince the interviewer of their honesty or to monitor the interviewer’s reactions, potentially reduce their gaze aversion.

Although liars reporting greater mental difficulty than truth-tellers, our study found no differences in illustrators and body part movements between liars and truth-tellers. This outcome contradicts the cognitive predictions. A possible explanation for this could be the insufficient cognitive load experienced by liars, as evidenced by previous studies. Such studies have increased the cognitive load on liars through interventions such as requiring participants to narrate events in reverse order or maintain eye contact while responding to questions, and found that the level of cognitive load indeed correlates with deception cues (Vrij et al., 2008, 2010). As for illustrators, the lack of difference could be due to their function in aiding verbal communication. In the mock interrogation of our study, there were no instances that necessitated participants to use illustrators to supplement their communication.

Our findings of differences in emotions and cognitive load, along with observable behaviors such as self-adaptors and the duration of gaze aversion between liars and truth-tellers, substantially enhance our understandings of deception cues, particularly in the context of Asia culture. Nevertheless, there are still several limitations in this study. First, the sample size of only 50 participants constrained our exploration to primarily examining the main effects of veracity, with gender and SES as control variables. This limited sample size hindered the exploration of interactive effects among veracity, gender, and SES, attributable to insufficient statistical power. Future research with larger samples would be beneficial to examine the effects of these variables on deception cues. Second, the measurement of participants’ psychological processes relied on self-report questionnaires administered after the mock interrogation. Participants’ assessments of their psychological states during the interrogation were based on retrospective recall, potentially introduce biases associated with memory. Future research could address this limitation by integrating physiological measures (e.g., heart rate variability, electrodermal activity) during the mock interrogation, coupled with post-interrogation self-report questionnaires, to capture participants’ psychological states. Third, while this study identified differences in self-adaptors and gaze aversion duration between liars and truth tellers, it did not reveal differences in other behavioral cues, including emotionally-linked behaviors such as blinks, and cognition-related cues such as illustrators and body part movements. This indicates the complexity of deception cues, which are subject to a multitude of factors (Vrij, 2008). Future investigations should aim to better control potential influences to facilitate the identification of deception cues.

## Conclusion

The present study presents compelling evidence of the psychological and behavioral distinctions between liars and truth-tellers. It highlights behaviors, including self-adaptors and duration of gaze aversion, may serve as potential indicators of deception. These insights contribute to our understanding of deception cues and may have practical implications in contexts like criminal interrogation.

## Data availability statement

The raw data supporting the conclusions of this article will be made available by the authors, without undue reservation.

## Ethics statement

The studies involving humans were approved by Northwest University Research Ethics Committee at Northwest University. The studies were conducted in accordance with the local legislation and institutional requirements. The participants provided their written informed consent to participate in this study.

## Author contributions

HeL: Conceptualization, Writing – original draft, Writing – review & editing. HS: Data curation, Writing – review & editing. ML: Data curation, Writing – review & editing. HaL: Conceptualization, Writing – review & editing.
