# Supplementary material for: Nonverbal cues to deception: insights from a mock crime scenario in a Chinese sample
Source: Front Psychol. 2024 Feb 9;15:1331653. doi: 10.3389/fpsyg.2024.1331653 (PMC10884279; doi:10.3389/fpsyg.2024.1331653)
Supplement: Supplementary file 1 [file Table_1.DOCX]

Questionnaire of Objective Socioeconomic Status

Please answer the following questions honestly.

1. What is your educational background?

1 = Literacy level is very low or illiterate

2 = Primary school

3 = Junior high school

4 = High school

5 = Associate degree

6 = Bachelor's degree

7 = Master's degree or above

1. What is your occupation?

1 = Temporary worker or unemployed

2 = Manual worker

3 = Skilled worker

4 = Ordinary office worker

5 = Ordinary management or technical staff

6 = Middle management or technical staff

7 = Senior management or technical staff

1. What is your average monthly income?

1 = Less than 2000 Yuan

2 = 2000-3000 Yuan

3 = 3000-4000 Yuan

4 = 4000-6000 Yuan

5 = 6000-8000 Yuan

6 = 8000-10000 Yuan

7 = Above 10000 Yuan
